# Machine learning assisted hepta band THz metamaterial absorber for biomedical applications

**DOI:** 10.1038/s41598-023-29024-x

**Published:** 2023-01-31

**Authors:** Prince Jain, Himanshu Chhabra, Urvashi Chauhan, Krishna Prakash, Akash Gupta, Mohamed S. Soliman, Md. Shabiul Islam, Mohammad Tariqul Islam

**Affiliations:** 1 Department of Mechatronics Engineering, Parul University, Vadodara, Gujarat India; 2 Department of Electronics and Communication Engineering, Parul University, Vadodara, Gujarat India; 3 Department of Electronics and Communication Engineering, SRM University, Amaravati, Andhra Pradesh India; 4 Division of Electronics and Communication Engineering, The LNM Institute of Information Technology (LNMIIT), Jaipur, India; 5 Department of Electrical Engineering, College of Engineering, Taif University, P.O. Box 11099, Taif, 21944 Saudi Arabia; 6 Department of Electrical Engineering, Faculty of Energy Engineering, Aswan University, Aswan, 81528 Egypt; 7 Faculty of Engineering (FOE), Multimedia University, Persiaran Multimedia, Cyberjaya, 63100 Selangor, Malaysia; 8 Department of Electrical, Electronic and Systems Engineering, Faculty of Engineering and Built Environment, Universiti Kebangsaan Malaysia, Bangi, Malaysia

**Keywords:** Metamaterials, Electronic devices, Nanophotonics and plasmonics, Sensors

## Abstract

A hepta-band terahertz metamaterial absorber (MMA) with modified dual T-shaped resonators deposited on polyimide is presented for sensing applications. The proposed polarization sensitive MMA is ultra-thin (0.061 *λ*) and compact (0.21 *λ*) at its lowest operational frequency, with multiple absorption peaks at 1.89, 4.15, 5.32, 5.84, 7.04, 8.02, and 8.13 THz. The impedance matching theory and electric field distribution are investigated to understand the physical mechanism of hepta-band absorption. The sensing functionality is evaluated using a surrounding medium with a refractive index between 1 and 1.1, resulting in good Quality factor (Q) value of 117. The proposed sensor has the highest sensitivity of 4.72 THz/RIU for glucose detection. Extreme randomized tree (ERT) model is utilized to predict absorptivities for intermediate frequencies with unit cell dimensions, substrate thickness, angle variation, and refractive index values to reduce simulation time. The effectiveness of the ERT model in predicting absorption values is evaluated using the Adjusted R^2^ score, which is close to 1.0 for *n*_min_ = 2, demonstrating the prediction efficiency in various test cases. The experimental results show that 60% of simulation time and resources can be saved by simulating absorber design using the ERT model. The proposed MMA sensor with an ERT model has potential applications in biomedical fields such as bacterial infections, malaria, and other diseases.

## Introduction

Metamaterial (MTM) is an artificial material with unique electromagnetic (EM) properties that are not available in nature^1,2^. Using the unusual EM properties of metamaterials, a lot of research has been done on antennas^3,4^, solar cells^5,6^, sensors^7–9^, and absorbers^10,11^ in the microwave to infrared frequency range. Metamaterial absorbers (MMAs) have received a lot of attention in the THz range because of their perfect absorption properties and potential applications in sensing, imaging, biotechnology, polarization conversion, and high-speed THz communications. Since Landy et al.^9^ demonstrated the first MMA, various types of MMAs have been proposed, including dual-^12^, triple-^13^, quad-^14^, penta-^15^, hepta-band^7^, and broadband^16^ absorbers. Among these, multi-band MMAs with high figure of merit (FOM) have received a lot of attention because they can be used to detect materials and hazardous gases^17,18^, and spectroscopic imaging^19^.

Several methods to increase absorption peaks have been proposed, such as stacking metallic resonators^16,20,21^ or incorporating resonators of various sizes in a single unit cell^22,23^. Recently, Wang et al.^24^ used four different sizes of metallic resonators to demonstrate quad-band MMAs. Additionally, dipolar resonance was used to propose triple-band, and quad-band absorbers based on triple-, and quad-square loop, respectively^22,25^. Wang et al. developed a triple-band THz MMA to attain multiband absorption at 0–3 THz^26^. In 2018, Janneh et al. presented a dual-band THz absorber consisting of a resonator on a polyimide spacer with a high Q-factor^27^. However, these approaches present manufacturing challenges at higher frequencies due to their larger size and thickness, making them impractical in practical applications^22,26,27^. Additionally, T-shaped^28^, ring-strip^29^, cave-ring^13^, and #-shaped^30^ MMAs also achieved multi-band absorption by integrating LC and dipolar resonances. The linewidth for these absorption devices is typically very large, ranging from one-tenth to one-fifth of the absorption frequency^31–34^. Since the absorption peak's linewidth is too wide, it cannot be used for practical sensor and detection applications. To achieve good sensing performance, the proposed absorption devices must have a narrow linewidth of the resonance, which is an important parameter when analyzing sensor applications^31–33^.

The proposed MMA consists of modified dual T-shaped resonators and ground plane spaced by a dielectric layer, resulting in multiple absorption peaks at 1.89, 4.15, 5.32, 5.84, 7.04, 8.02, and 8.13 THz. The electric field distribution is analyzed to understand the absorption mechanism, which suggests a physical mechanism of LC and crossed dipole resonance resulting in size reduction. Furthermore, to investigate the absorption dependence, parametric analysis was carried out using dielectric thickness and unit cell dimension. The proposed device has a simple structural design, less thickness, compact size, and narrow linewidth properties, suggesting sensing and detection applications. Table 1 compares the performance of nine relevant works with our work which clearly shows that the FOM and Q in our design outperform the previous work in Ref^31–33,35–42^. The fourth peak of absorption response has FOM of 44, full width at half maximum (FWHM) value of 0.05 THz, peak sensitivity of 2.2 THz/RIU, and Q-factor value of 117. Furthermore, Extreme randomized tree (ERT) model is used to predict absorption values for intermediate frequencies to reduce 60% of simulation time and resources. The proposed MMA has the potential to be implemented in a variety of biomedical sensing applications, including the detection of glucose and malaria due to its high Q-factor and narrow FWHM.

**Table 1:** Comparison of FOM and Q value of the proposed device with the previously reported terahertz MMA.

References	FOM	Q
^35^	1.5	40
^33^	0.85	8.5
^31^	2.94	22.1
^36^	0.7	7.8
^37^	2.4	49.5
^38^	0.4	11.9
^39^	–	7.68
^40^	2.8	16.3
^41^	13.88	38.407
This paper	44	117

## Design and simulation

**Figure 1:**
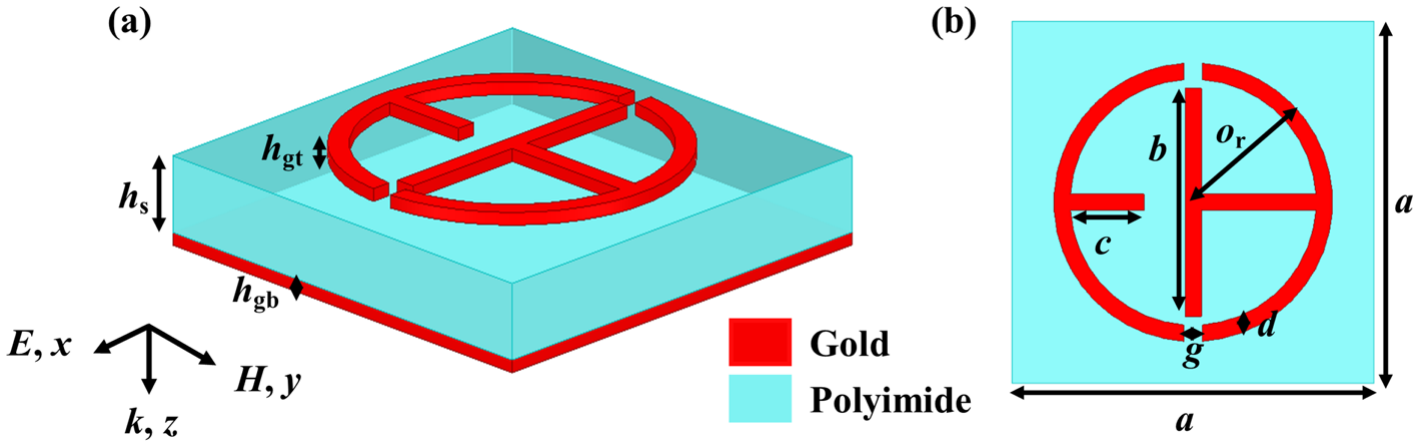
(**a**) Perspective view and (**b**) Top view of the proposed metamaterial absorber (MMA).

Figure 1a,b depict a perspective and top view of a three-layer hepta-band metamaterial **absorber. The top metallic layer is composed of two modified T-shaped resonators, while the bottom layer consists of a continuous metallic plane, and both layers are made of gold with a conductivity of 4*.*09 × 10^7^ S*/*m. The middle layer is composed of polyimide with a dielectric constant of 3 (1 + *j*0.06). Table 2 illustrates the optimized dimensional parameters of a metamaterial structure. At the lowest operational frequency, the compactness and thickness of the absorber is 0.21λ and 0.061λ, respectively. The absorption is $1-{\left|{S}_{11}\right|}^{2}-{\left|{S}_{21}\right|}^{2}$, where *S*_11_ and *S*_21_ represents the reflection and transmission coefficients, respectively. Here, ${\left|{S}_{11}\right|}^{2}={\left|{S}_{11,xy}\right|}^{2}+{\left|{S}_{11,xx}\right|}^{2}$ where *S*_11,*xy*_ and *S*_11,*xx*_ represent the reflection of cross-polarized and co-polarized EM waves, respectively^8^. The maximum absorption can be achieved when *S*_11_ and *S*_21_ are reduced to zero. The transmission coefficient can be reduced to zero (*S*_21_ = 0) since the thicknesses of the ground plane is greater than skin depth. Furthermore, geometric parameters such as the thickness and periodic dimension of the gold resonator must be optimized in order to reduce the reflection coefficient.

**Table 2:** Geometrical parameters of a metamaterial structure.

Parameters	Values (µm)	Description
*h*_gt_	0.2	Thickness of top metallic layer
*h*_s_	9.7	Thickness of dielectric layer
*h*_gb_	0.5	Thickness of bottom metallic layer
*o*_r_	14.8	Radius of circular ring
*g*	2	Split in circular ring
*d*	1.8	Width of circular ring
*c*	7.8	Length of inner rectangular bar
*b*	24	Length of vertical rectangular bar
*a*	34.4	Lattice period of the absorber

The proposed MMA is simulated using the ANSYS High Frequency Structure Simulator (Version 2022 R1) software with Floquet periodic port in the z-direction and periodic boundary conditions in x- and y-directions. During simulation, the EM waves that are polarized along the y-axis are often incident on the surface of the absorber along the z-axis. The fabrication of the MMA can be achieved using the following steps. First, the middle dielectric layer of the metamaterial absorber could be created by evenly spin coating the polyimide material with an optimized thickness on the Au film. Second, the middle dielectric layer for the top Au patterned array could be transferred using the spin-coating, exposure, development, evaporation, and lift-off technologies^43^.

## Results and discussions

**Figure 2:**
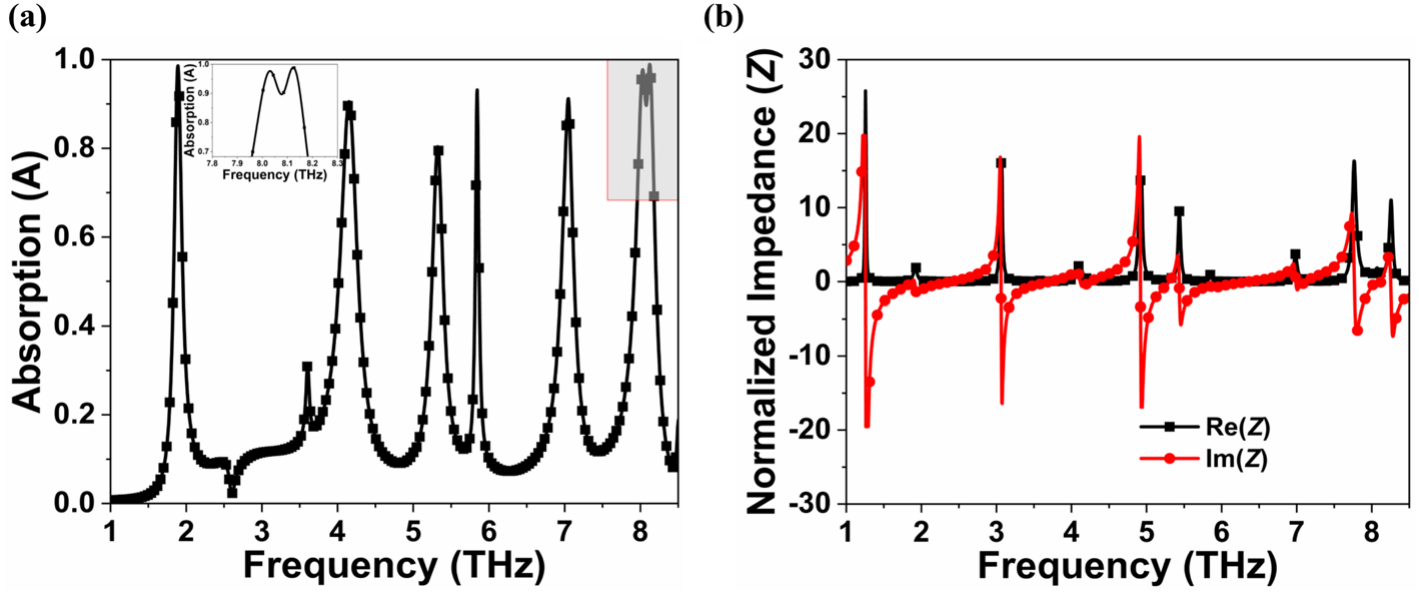
(**a**) Absorption spectra with its inset of *f*_6_ and *f*_7_ resonating frequencies and (**b**) Real and imaginary normalized impedance of the proposed MMA.

The proposed MMA exhibits multiple absorption peaks at 1.89 (*f*1), 4.15 (*f*2), 5.32 (*f*3), 5.84 (*f*4), 7.04 (*f*5), 8.02 (*f*6) and 8.13 THz (*f*7) with absorption coefficients of 98.75, 90.39, 80.40, 93.03, 91.2, 97.23 and 98.25%, respectively as shown in Fig. 2a. The FWHM bandwidth at *f*4 is 0.05 THz and quality factor (Q = *f*c/FWHM, where *f*c represents the resonance's center frequency) is 117, which is exceptional to already reported absorbers^44^. The frequency mode *f*4 has a Q value of 117, which is approximately 8.7, 7.3, 3.3, and 2.8 times greater than the other frequency modes *f*1, *f*2, *f*3, and *f*5, respectively. The Quality factor is known to be a critical parameter for evaluating frequency mode performance, with a higher Q value indicating that the device is more effective in sensing applications.

**Figure 3:**
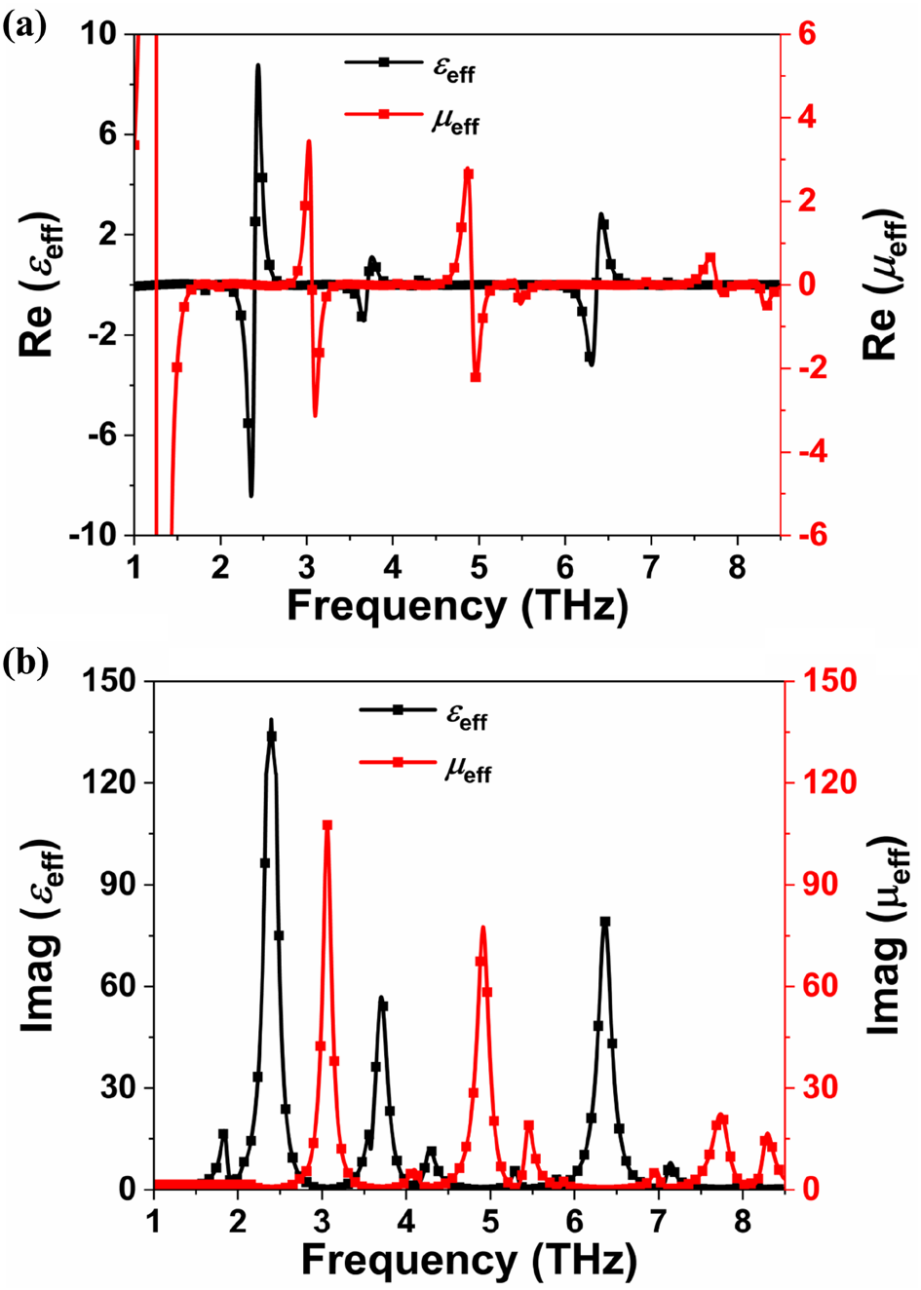
(**a**) Real parts of *ε*_eff_ and *μ*_eff_ and (**b**) Imaginary parts of *ε*_eff_ and *μ*_eff_ of the proposed absorber.

The MMA impedance must be equal to the free space impedance to achieve perfect absorption, and for that, the real and imaginary normalized impedances should be close to one and zero, respectively^10,26^. The obtained *Z* is 1.02 + *j*0.23, 1.02-*j*0.66, 0.42 + *j*0.28, 0.83-*j*0.47, 0.87-*j*0.59, 1.22-*j*0.26 and 1.22 + *j*0.19 at 1.89, 4.15, 5.32, 5.84, 7.04, 8.02 and 8.13 THz, respectively, as demonstrated in Fig. 2 (b). Effective permittivity (*ε*_eff_) must equal effective permeability (*µ*_eff_) to achieve unity impedance, as $Z=\sqrt{{\mu }_{\mathrm{eff}}/{\varepsilon }_{\mathrm{eff}}}$. Real values of *ε*_eff_ and *µ*_eff_ must equal imaginary values in order to obtain perfect absorption, which is an ideal but challenging condition to achieve. As a result, the MMA's absorptivity is less than 100% since the normalized impedance will never be the same as the characteristic impedance. Figure 3a,b show the *ε*_eff_ and *µ*_eff_ of the proposed MMA, which were calculated using the S parameter retrieval method. Table 3 shows that the real and imaginary values of *ε*_eff_ and *µ*_eff_ are found to be equal because structure impedance equals free space impedance at perfect absorption.

**Table 3:** Comparison of absorption rate, normalized impedance and EM parameters.

Frequency (THz)	Absorption Rate (%)	Re (*Z*)	Im (*Z*)	Re (*ε*_eff_)	Re (*µ*_eff_)	Im (*ε*_eff_)	Im (*µ*_eff_)
1.89	98.75	1.02	0.23	−0.013	0.019	3.74	1.51
4.15	90.39	1.02	−0.66	0.02	−0.03	4.78	1.14
5.32	80.40	0.42	0.28	0.13	0.03	3.14	1.08
5.84	93.03	0.83	−0.47	0.01	−0.01	2.18	1.29
7.04	91.2	0.87	−0.59	−0.002	0.001	1.32	1.47
8.02	97.23	1.22	−0.26	−0.001	0.003	1.00	1.50
8.13	98.25	1.22	0.19	−0.002	0.002	0.97	1.49

**Figure 4:**
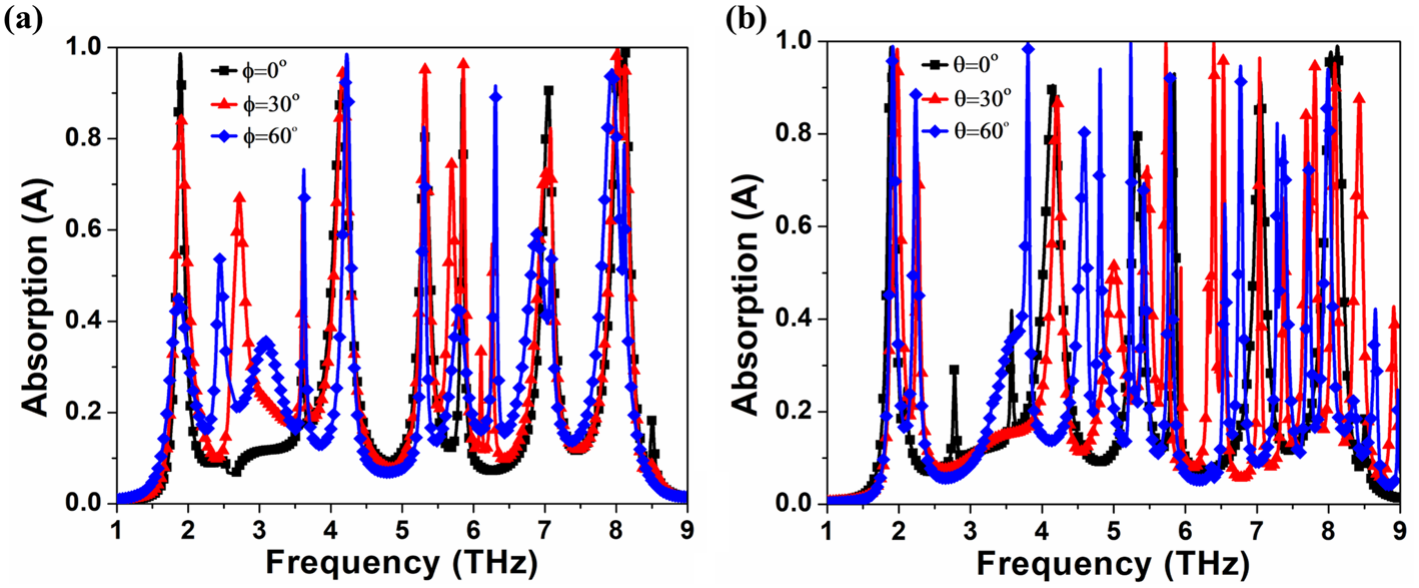
Simulated absorption response at various (**a**) polarization angles (*ϕ*) from 0 to 60° and (**b**) incident angles (*θ*) from 0 to 60°.

**Figure 5:**
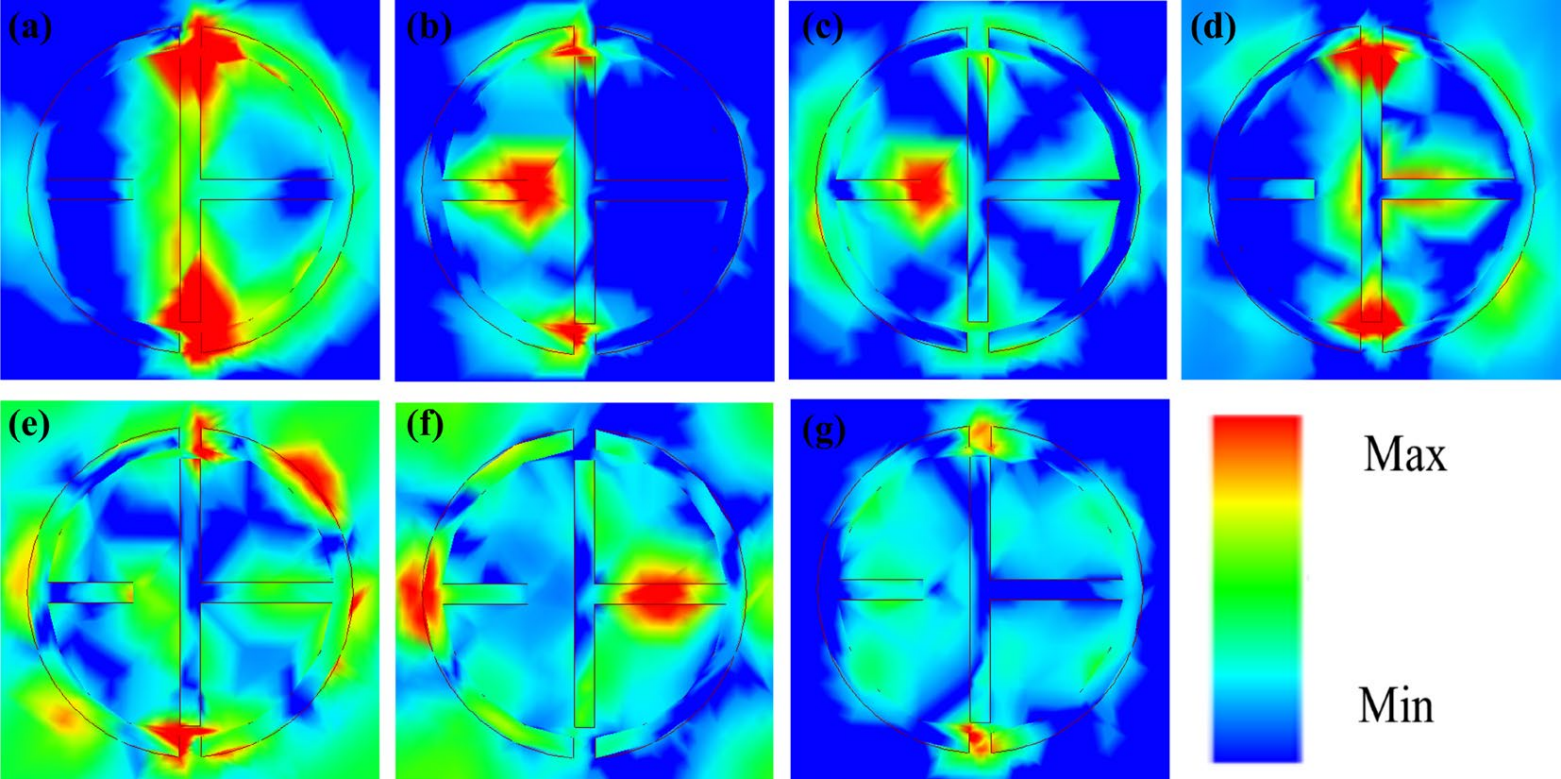
Electric field distribution on the top plane at 1.89 (**a**), 4.15 (**b**), 5.32 (**c**), 5.84 (**d**), 7.04 (**e**), 8.02 (**f**) and 8.13 THz (**g**).

Figure 4a depicts the absorption response at various polarization angles, demonstrating that the asymmetric structure of the absorber makes it polarization sensitive. Such properties have a wide range of potential applications, including sensing, detection, and optoelectronics. Furthermore, the proposed MMA is demonstrated with different incident angles, implying that the proposed absorber is sensitive to incidence angles as shown in Fig. 4b, similar to previously reported work^44^. The e-field distribution is analyzed at 1.89, 4.15, 5.32, 5.84, 7.04, 8.02, and 8.13 THz to better understand the absorption mechanism, as illustrated in Fig. 5. The e- field is primarily accumulated on the inner side of the resonator which is attributed as LC or fundamental resonance, as shown in Fig. 5a,d,e. The e-field distributions are focused at the resonator's edges, which is related to the structure's crossed dipole or higher-order resonance occurring at higher frequencies because the size of the proposed structure is greater than a multiple of a half-wavelength of the resonant modes^45^, as shown in Fig. 5b,c,f. The e-field distribution can be found inner and outer sides of the resonator due to the structure's combination of LC and crossed dipole resonance as shown in Fig. 5e,g. These results suggest a novel method for designing a multi-band MMA that incorporates various resonance modes.

**Figure 6:**
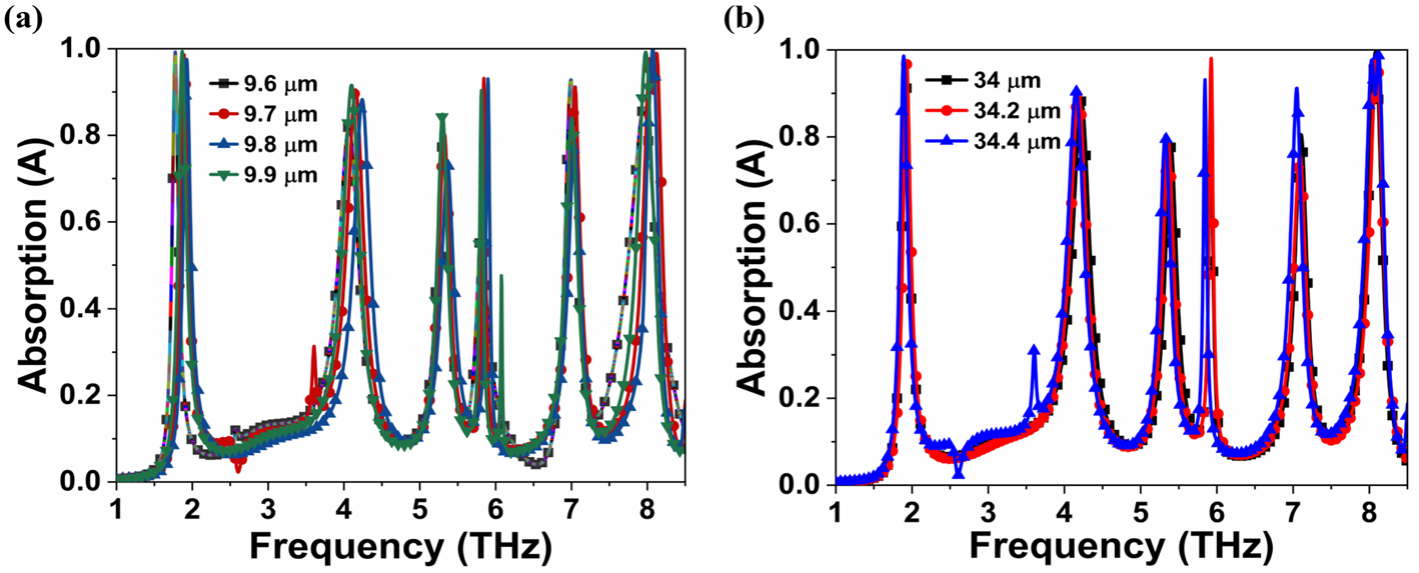
Absorption response at different (**a**) substrate thickness (*h*_s_) from 9.6 to 9.9 μm and (**b**) unit cell dimension (*a*) from 34 to 34.4 μm.

As shown in Fig. 6a,b, parametric analysis is performed with the substrate thickness (*h*_s_) and unit cell dimension (*a*) to determine their effect on the absorption response. The inverse effects of *h*_s_ on resonance frequencies can be estimated using transmission phase:

1 $$\alpha =\frac{4{h}_{s}\sqrt{{\varepsilon }_{r}-{sin}^{2}\theta }}{\uplambda }$$

where *ε*_r_ and λ is the dielectric constant and wavelength, respectively. The ratio *h*_s_/*λ* is remain fixed with constant value of *ε*_r_, *α*, and *θ*. As a result, the relationship between the resonating frequency and the substrate thickness is inversely proportional. However, *a* and λ are directly proportional to each other resulting in inverse effects on resonance frequencies. As a result, the absorption response shows red shift phenomenon as the *h*_s_ and *a* increases^15^. Table 4 compares previously reported studies^10,11,13,15,28,46–48^ with proposed MMA in terms of unit cell size, thickness, and resonating frequencies, indicating that the proposed MMA has seven absorption peaks with compact metallic structure, which is superior to previously reported MMA.

**Table 4:** Comparison of the previously reported THz MMAs with the proposed MMA.

Ref	Lowest frequency (THz)	Unit Cell size (μm)	Dielectric Thickness (μm)	Resonances
^28^	1.12	0.283 λ_0_	0.029 λ_0_	Quad
^46^	1.85	0.431 λ_0_	0.043 λ_0_	Quad
^13^	6.53	0.435 λ_0_	0.07 λ_0_	Triple
^15^	3.95	0.466 λ_0_	0.066 λ_0_	Penta
^11^	3.5	0.35 λ_0_	0.05 λ_0_	Dual
^10^	0.48	0.432 λ_0_	0.084 λ_0_	Single
^47^	1.60	0.66 λ_0_	0.13 λ_0_	Quad
^48^	15.68	0.418 λ_0_	0.031 λ_0_	Triple
Proposed work	2.33	0.21λ_0_	0.061λ_0_	Hepta

### Refractive index sensing

**Figure 7:**
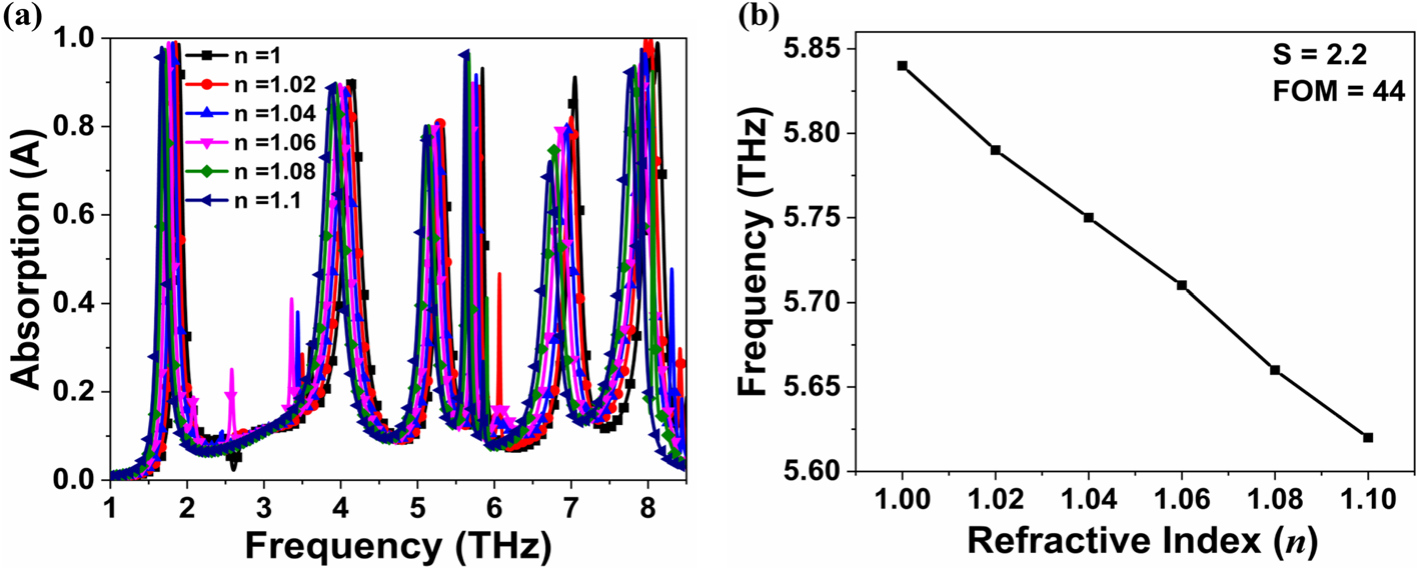
(**a**) Absorption response with different refractive index (*n*) and (**b**) relation between frequency and refractive index.

In comparison to the other resonance peaks, the fourth resonance peak has a narrow FWHM and a high Q, as previously discussed. In Fig. 7, the frequency dependence is demonstrated by changing the refractive index (*n*) of the surrounding environment from 1.00 to 1.10. The total frequency shift of *f*_1_, *f*_2_, *f*_3_, and *f*_5_ were found to be approximately 0.23, 0.28, 0.22, and 0.32 THz, respectively, as shown in Fig. 7a. The dielectric constant (*ɛ*_r_) is proportional to the *n* with *ɛ*_r_ = *n*^2^, thus increase in *n* increases the capacitance of the over layer, which is inversely proportional to resonance frequency^49^. As a result, Fig. 7b depicts the inverse relationship between the resonance frequency (*f*_4_) and the refractive index. The sensitivity (*S*) is *Δf*/*Δn* (THz/RIU) where *Δn* and *Δf* represent change in the refractive index and frequency, respectively^49–51^. The *S* values for frequency modes *f*_1_, *f*_2_, *f*_3_, *f*_4_, and *f*_5_ were found to be 2.3, 2.8, 2.2, 2.2, and 3.2 THz/RIU, respectively. FOM is also an important parameter to compare sensing performance for different types of sensors which is calculated as S/FWHM. The FOM values of the resonating frequency *f*_1_, *f*_2_, *f*_3_, *f*_4_, and *f*_5_ are 15.33, 9.33, 12.22, 44, and 20, respectively. The FOM of resonating frequency *f*_4_ is approximately 2.9, 4.7, 3.6, and 2.2 times that of the other resonating frequency, *f*_1_, *f*_2_, *f*_3_, and *f*_5_, respectively. It is clear from the results that proposed absorber has potential applications in a variety of fields, including solar energy, stealth technology, sensing, and detection.

### Application in biochemical sensing

**Figure 8:**
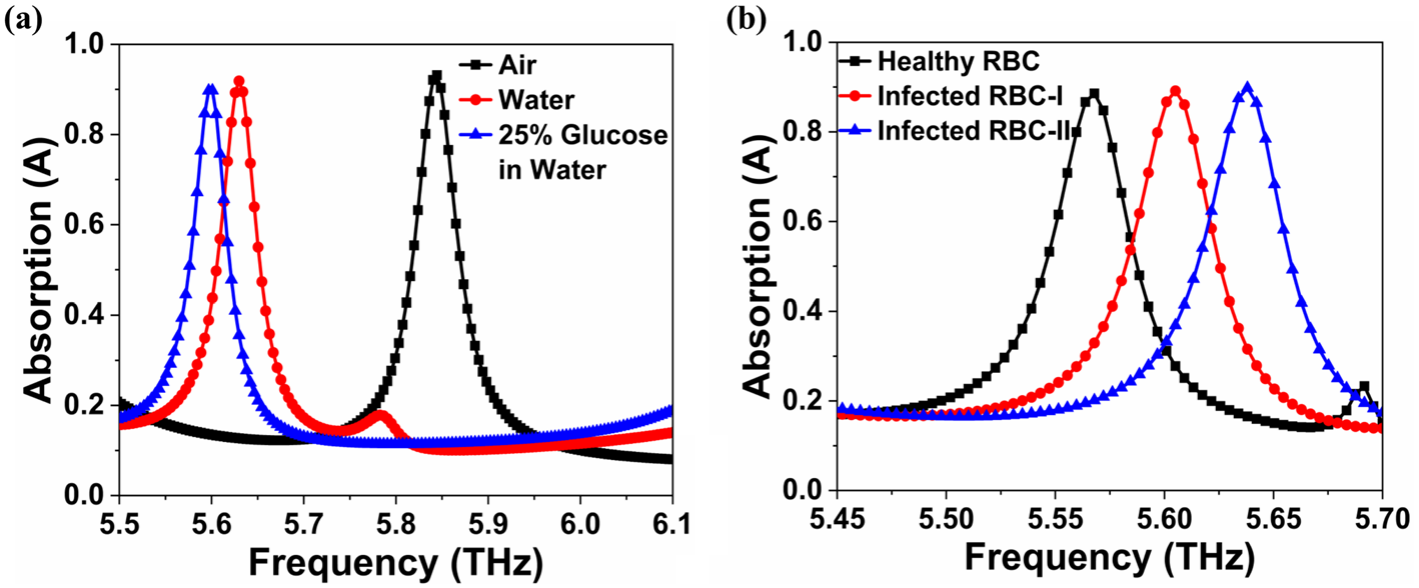
Effect on absorption response with the sensing of (**a**) Glucose and (**b**) Malaria.

Glucose is a vital biochemical that powers essential metabolic functions in the human body. Therefore, we can assess the suitability of the proposed structure as a biochemical sensor by simulating two different scenarios for detecting the presence of bio-molecules. Water has a refractive index of *n*_w_ = 1.3198 and 25% glucose in water has a refractive index of *n*_gw_ = 1.3594^41,52^. The fourth peak has a sensitivity of 2.1 THz/RIU in water and 4.7 THz/RIU in water with 25% glucose, ensuring the MMA's enhanced detection capabilities, as shown in Fig. 8a. Future MMA fabrication could pave the way for more sensitive biomolecule detection^53^.

### Application in detection of malaria

Rapid malaria screening is crucial because 250 million people worldwide are infected each year^41,54^. The healthy red blood cell (also known as an RBC) has a refractive index of 1.399. Furthermore, as malaria progresses, the refractive indices of infected RBCs are 1.373 and 1.383 in the schizont and trophozoite phases, respectively. The proposed sensor can detect various stages of malaria, as shown in Fig. 8b. The proposed sensor achieved a high sensitivity value of 2.5 and 2.7 THz/RIU for infected RBC-I and II, respectively. Recent metamaterial-based biosensors show good agreement between simulation and measurement results^55^. This latest study confirms that the proposed absorber/sensor will perform as expected if experimentally implemented in the future.

### Prediction of absorption coefficient using machine learning technique

In this section, the purpose of regression models for the simulation process is briefly discussed, along with how regression models can reduce the time and resource requirement to simulate the effective absorber design by 40%, 50%, or 60%. Regression analysis is an effective approach used to determine dependent parameter values based on independent parameter values. In the present work, frequency is an independent parameter in absorber design, whereas absorption coefficient is a dependent parameter. The designing process of a complex structure takes more time and resources to simulate the experimental design. These issues can be effectively handled with the application of machine learning (ML) approaches for regression problems and can identify the missing parameter values as well. The following three stages can be utilized to fix this issue using ML-based regression analysis methodologies.

Step 1: Simulate the absorber's design by increasing the frequency's step size value.

Step 2: Using simulated data, train the ML-based regression model.

Step 3: Predict the absorptivity of intermediate frequencies using the trained regression model.

### Extreme randomized tree (ERT) regression model

In the present study, the regression analysis has been done using ERT-Model which is constructed by a binary recursive partitioning mechanism to enhance the prediction accuracy. The motivation to utilize this technique is that the explicit randomization of the cut-off points and attribute is aggregated with ensemble averaging, thus it can reduce variance more strongly than the weaker randomization schemes. The technique has two vital parameters *i.e.,* number of attributes randomly selected at each node (*K*) and the minimum sample size for splitting a node (*n*_min_). Parameter *K* determines the attributes strength employed to predict the target, such that *K* = 1……*p*, where *p* is the number of independent parameters employed to predict target parameter. In regression problems, larger value of K is preferred for enhanced accuracy^56^. In this work, total six attributes *i.e.*, substrate thickness (*h*_s_), unit cell dimension (*a*), incident angle (*θ*), polarization angle (*ϕ*), refractive index (*n*) and frequency (*f*) are chosen for predicting the absorption coefficient. For a wide range of regression applications, the value of *n*_*min*_ should range between 2 and 10^56^.

In ERT model, a collection of “*m*” unpruned regression trees (*RT*1, *RT*2….*RT*m) is constructed. The predictions of the trees are aggregated to yield the final prediction by computing arithmetic average which is used to amalgamate the outcomes of each regression tree as indicated by the given equation:

2 $$\mathrm{predicted\,output}=\sum_{j=1}^{m}{RT}_{j}(x)$$

where *x* denotes the independent parameter value and *m* denotes the number of trees. Several performance indices can examine the trained regression model accuracy such as R-Square Score (R^2^S) and Adjusted R Square Score (Adj-R^2^S), Integral absolute error (IAE), Mean Absolute Percentage Error (MAPE) and Mean Squared Error (MSE), etc. Out of these, R^2^S and Adj-R^2^S are often used metrics to assess how accurately a trained regression model predicts outcomes. These considered indices can be computed using Eqs. (3) and (4) as follows:

3 $${R}^{2}S=1-\frac{\sum_{i=1}^{N}{\left(Predicted\,Valu{e}_{i}-Actual\,Valu{e}_{i}\right)}^{2}}{\sum_{i=1}^{N}{\left(Actual\,Valu{e}_{i}-Average\,Target\,Valu{e}_{i}\right)}^{2}}$$

4 $${Adj-R}^{2}S=1-\frac{(1-{R}^{2}S)(N-1)}{N-p-1}$$

where ‘*N*’ is the number of samples used to test the regression model.

In this work, rigorous analysis has been carried out for predicting the absorption coefficient value using designed ERT regression model. The effectiveness of the ERT model prediction is assessed under three different test cases i.e. TC-40, TC-50, and TC-60. In TC-40, two separate subsets of the simulation-generated data are used. In one subset, 40% of the simulation records were chosen at random, and trained for the ERT regression model, while 60% of the simulation records were chosen for another subset for the model's predictive accuracy. Two equal-sized, non-overlapping subsets of simulation records are created from the data generated during the simulation of TC-50. From the simulation data, one subset comprises even numbers of rows, and another contains odd numbers of rows. Any partition can be used to train the ERT regression model, and others can be used to evaluate the model's predictive power. Additionally, the data produced during simulation in TC-60 is divided into two distinct categories. One subgroup contains 60% randomly selected simulation records, while the other contains 40%. One subset is utilized to train the ERT-Regression model, and the other subset is used to assess the model's predictive ability.

In all considered test cases TC-40, TC-50, and TC-60, machine learning models are used to determine how significantly simulation resource consumption can be minimized. If regression models are effective at predicting absorption values for TC-40, it shows that an ERT model trained with 40% of the simulation records will be able to predict absorption values for the remaining 60% of records, saving 60% of the simulation time and resources. Additionally, if the TC-60 regression model's prediction accuracy is high, it can be concluded that the ERT model trained with 60% of the simulation records can accurately predict absorption values for the remaining 40% of records, resulting in a 40% reduction in simulation time and resource needs.

**Figure 9:**
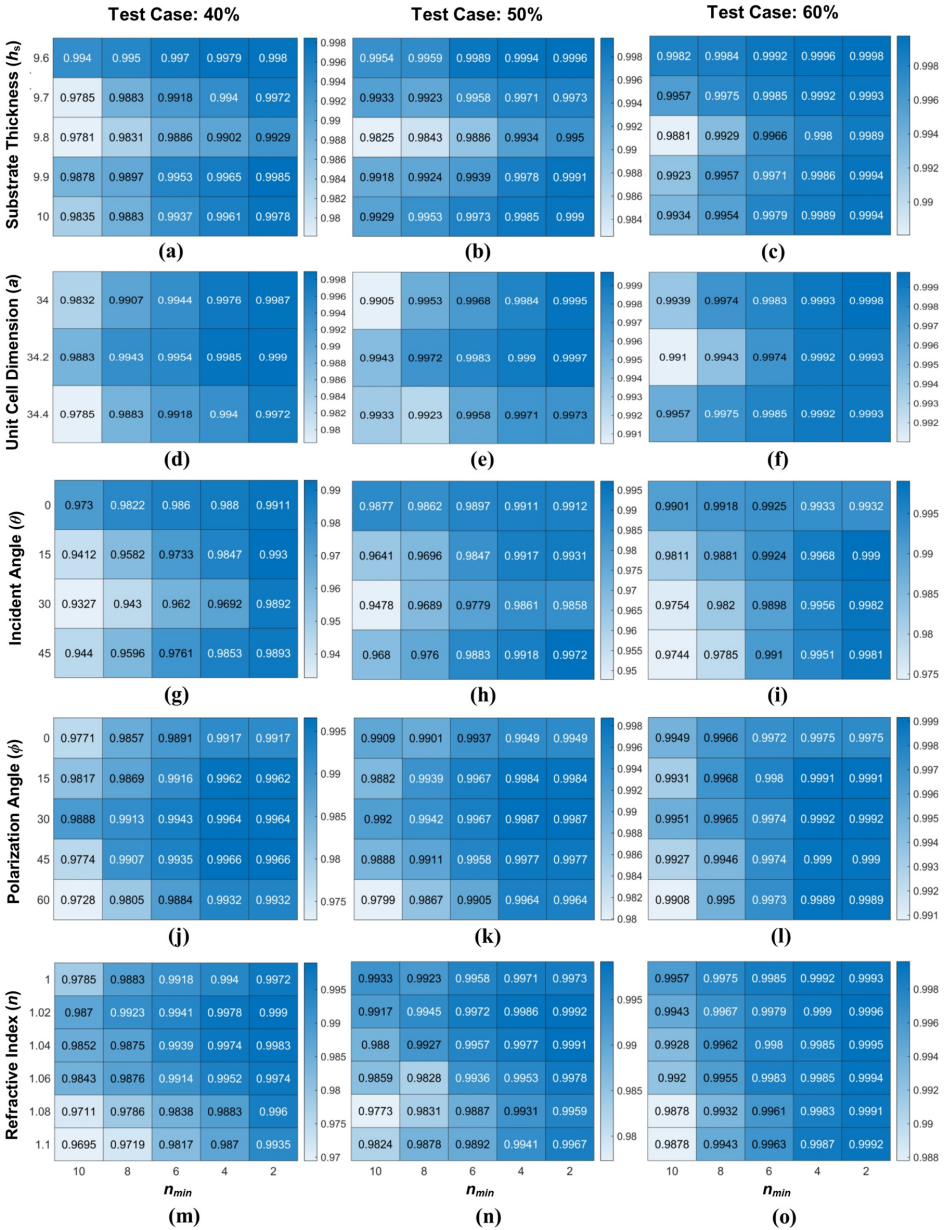
Heat map indicating Adjusted R^2^ Score of ERT model using numerous combinations of (**a**–**c**) substrate thickness, (**d**–**f**) unit cell dimension, (**g**–**i**) incident angle, (**j**–**l**) polarization angle and (**m**–**o**) refractive index for different test case 40%, 50%, and 60%. Images were produced using MATLAB (R2022b, https://www.mathworks.com/).

Figure 9 shows the heat map indicating Adj-R^2^ Score of ERT model using various combinations of attributes where *n*_min_ varies from 2 to 10. Figure 9a–c shows the Adj-R^2^S for different values of *h*s for test cases 40%, 50%, and 60% respectively. Similarly, the impact of other attributes (*a*, *ϕ*, *θ* and *n*) on Adj-R^2^S have been examined under considered test cases of 40%, 50%, and 60% which is depicted in Fig. 9d–o. The Adj-R^2^ score values approach to unity indicates high prediction accuracy with minimum prediction error^57,58^. It is clear from heat maps that in all the cases the most preferable Adj-R^2^S values are obtained when the smoothing strength *n*_min_ = 2.

**Figure 10:**
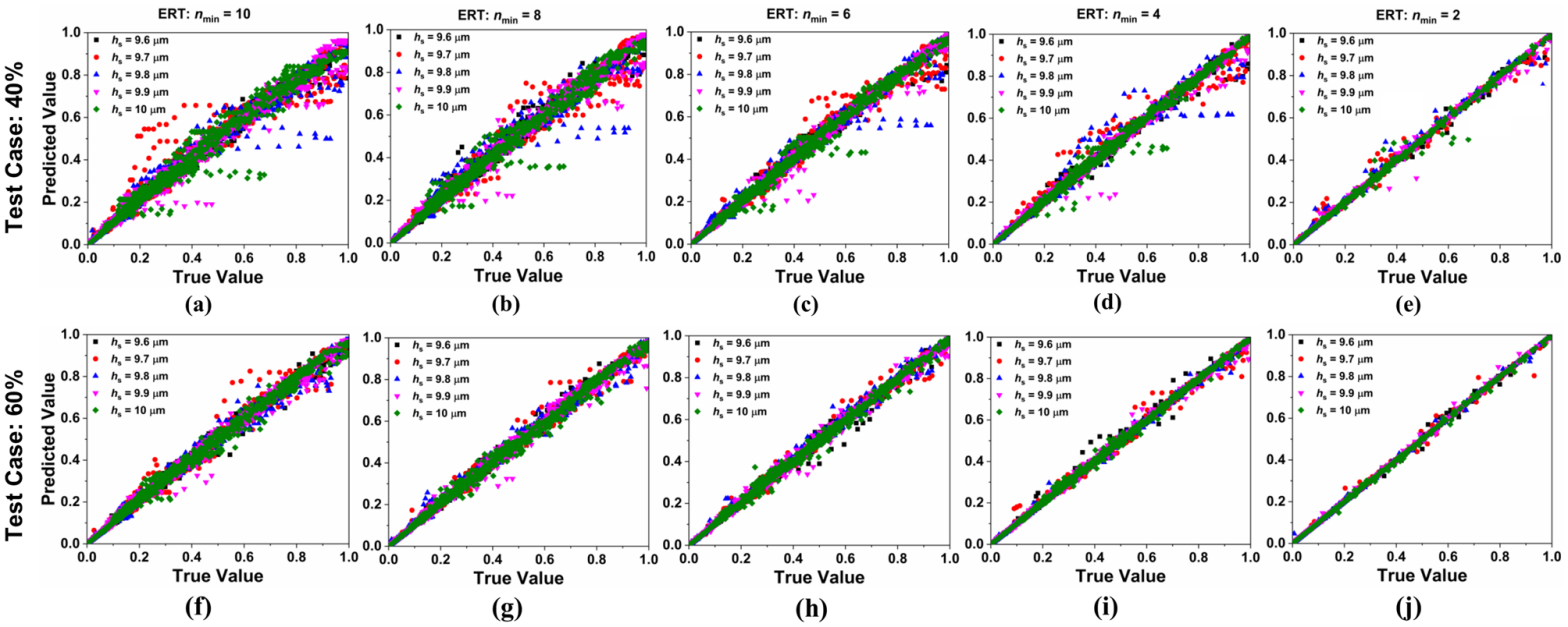
Values of absorption predicted by ERT model vs simulated values of absorption for various substrate thickness (*h*_s_) (**a**) *n*_min_ = 10 for test case (TC)- 40 (**b**) *n*_min_ = 8 for TC- 40 (**c**) *n*_min_ = 6 for TC- 40 (**d**) *n*_min_ = 4 for TC- 40 (**e**) *n*_min_ = 2 for TC- 40 (**f**) *n*_min_ = 10 for TC- 60 (**g**) *n*_min_ = 8 for TC- 60 (**h**) *n*_min_ = 6 for TC- 60 (**i**) *n*_min_ = 4 for TC- 60 and (**j**) *n*_min_ = 2 for TC- 60.

**Figure 11:**
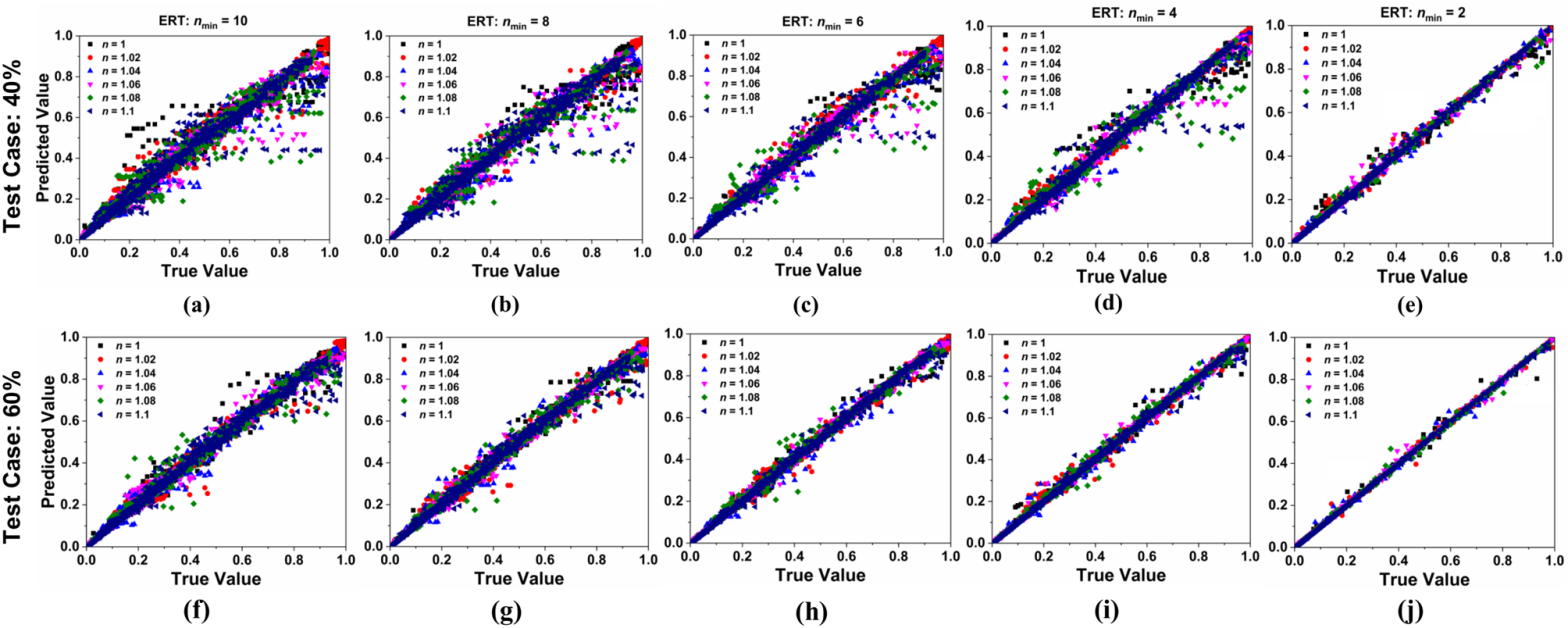
Values of absorption predicted by ERT model vs simulated values of absorption for various refractive index (*n*) values (**a**) *n*_min_ = 10 for TC-40 (**b**) *n*_min_ = 8 for TC- 40 (**c**) *n*_min_ = 6 for TC- 40 (d) *n*_min_ = 4 for TC- 40 (**e**) *n*_min_ = 2 for TC- 40 (**f**) *n*_min_ = 10 for TC- 60 (**g**) *n*_min_ = 8 for TC- 60 (**h**) *n*_min_ = 6 for TC- 60 (**i**) *n*_min_ = 4 for TC- 60 and (**j**) *n*_min_ = 2 for TC- 60.

Figure 10a–e shows the scatter plots of predicted absorption values by ERT model vs simulated absorption values for varying substrate thickness from 9.6 µm to 10 µm for different values of *n*_min_ under test case TC-40. Under similar conditions of *h*_s_ and *n*_min_, the scattered plot of prediction vs true value for TC-60 is shown in Fig. 10f–j. Results show that the predicted value approaches to true value when *n*_min_ is 2. Moreover, Fig. 11a–e shows the scatter plots of predicted absorption values by ERT regression model vs simulated absorption values for varying refracting index from 1 to 1.1 for different values of *n*_min_ under test case TC-40. Moreover, for similar refractive index and *n*_min_ conditions (as in Fig. 11a–e). The scattered plot of prediction values vs true value for TC-60 is shown in Fig. 11f–j. Results show that in all the cases the most preferable predicted values are obtained when the smoothing strength *n*_min_ = 2.

## Conclusions

For terahertz applications, a hepta-band MMA comprised of modified dual T-shaped resonators deposited on polyimide is presented. Multiple absorption peaks with absorptivities greater than 80% can be found at 1.89, 4.15, 5.32, 5.84, 7.04, 8.02, and 8.13 THz. The hepta-band absorption is primarily caused by the combination of the metallic resonator's dipolar response and LC resonance, as explained by examining the e-field distribution. The proposed MMA is suitable candidate for bio-medical applications due to its Q value of 117, FOM of 44 and high sensitivity of 4.72 THz/RIU. Furthermore, a machine learning assisted ERT regression model is used to learn absorber behavior and predict absorption values for intermediate frequencies. The Adjusted R^2^ score was close to 1.0 for *n*_min_ = 2, demonstrating the prediction efficiency of the ERT model in estimating absorption values in various Test Cases. The experimental results show that using the ERT model to simulate absorber design can reduce simulation time and resource requirements by 60%. The proposed MMA sensor designed with an ERT model is applicable in biomedical applications for the detection of malaria and glucose.

## Data Availability

All other data that support the findings of this study are available from the corresponding author upon reasonable request.

## References

[1] Shelby RA, Smith DR, Schultz S (2001). Experimental verification of a negative index of refraction. Science.

[2] Cai W, Chettiar UK, Kildishev AV, Shalaev VM (2007). Optical cloaking with metamaterials. Nat. Photonics.

[3] Jain P. *et al.* I-shaped metamaterial antenna for X-band applications. *In: Progress in Electromagnetics Research Symposium,* 2017 10.1109/PIERS.2017.8262229.

[4] Jain P. *et al.* T-shaped resonator for x-band applications. *In: IEEE MTT-S International Microwave and RF Conference, IMaRC,* 2018. 10.1109/IMaRC.2017.8449678.

[5] Wang Y (2012). Metamaterial-plasmonic absorber structure for high efficiency amorphous silicon solar cells. Nano Lett..

[6] Zhang X, Liu Z (2008). Superlenses to overcome the diffraction limit. Nat. Mater..

[7] Jain P (2022). Design of an ultra-thin hepta-band metamaterial absorber for sensing applications. Opt. Quant. Electron..

[8] Jain P (2020). Ultra-thin metamaterial perfect absorbers for single/dual/multi-band microwave applications. IET Microw. Antennas Propag..

[9] Landy NI, Sajuyigbe S, Mock JJ, Smith DR, Padilla WJ (2008). Perfect metamaterial absorber. Phys. Rev. Lett..

[10] Yan D (2019). Tunable all-graphene-dielectric single-band terahertz wave absorber. J. Phys. D: Appl. Phys..

[11] Zhang Y (2019). Dual-band switchable terahertz metamaterial absorber based on metal nanostructure. Results Phys..

[12] Jain P. *et al.* Dual band graphene based metamaterial absorber for terahertz applications. *In: IEEE 13th Nanotechnology Materials and Devices Conference, NMDC,* 2019. 10.1109/NMDC.2018.8605833.

[13] Huang X, Lu C, Rong C, Liu M (2018). Wide-angle perfect metamaterial absorbers based on cave-rings and the complementary patterns. Opt. Mater. Express..

[14] Jain P (2022). Quad-band polarization sensitive terahertz metamaterial absorber using Gemini-shaped structure. Results Opt..

[15] Zhang Y (2019). Five-band terahertz perfect absorber based on metal layer–coupled dielectric metamaterial. Plasmonics.

[16] Ding F, Cui Y, Ge X, Jin Y, He S (2012). Ultra-broadband microwave metamaterial absorber. Appl. Phys. Lett..

[17] Senesac L, Thundat TG (2008). Nanosensors for trace explosive detection. Mater. Today.

[18] Ritari T (2004). Gas sensing using air-guiding photonic bandgap fibers. OSA Trends Opt. Photon. Ser..

[19] Rodrigues SP, Lan S, Kang L, Cui Y, Cai W (2014). Nonlinear imaging and spectroscopy of chiral metamaterials. Adv. Mater..

[20] Tran CM (2019). Creating multiband and broadband metamaterial absorber by multiporous square layer structure. Plasmonics.

[21] He XJ (2015). Broadband and polarization-insensitive terahertz absorber based on multilayer metamaterials. Opt. Commun..

[22] Wang BX, Zhai X, Wang GZ, Huang WQ, Wang LL (2015). Design of a four-band and polarization-insensitive terahertz metamaterial absorber. IEEE Photon. J..

[23] Jain P (2021). An ultrathin compact polarization-sensitive triple-band microwave metamaterial absorber. J. Electron. Mater..

[24] Wang GD, Liu MH, Hu XW, Kong LH, Cheng LL, Chen ZQ (2014). Multi-band microwave metamaterial absorber based on coplanar Jerusalem crosses. Chin. Phys. B.

[25] Shen X (2011). Polarization-independent wide-angle triple-band metamaterial absorber. Opt. Express.

[26] Wang BX, Wang GZ (2017). New type design of the triple-band and five-band metamaterial absorbers at terahertz frequency. Plasmonics.

[27] Janneh M, De Marcellis A, Palange E, Tenggara AT, Byun D (2018). Design of a metasurface-based dual-band terahertz perfect absorber with very high Q-factors for sensing applications. Opt. Commun..

[28] Meng HY, Wang LL, Zhai X, Liu GD, Xia SX (2018). A simple design of a multi-band terahertz metamaterial absorber based on periodic square metallic layer with T-shaped gap. Plasmonics.

[29] Zhao L, Liu H, He Z, Dong S (2018). Theoretical design of twelve-band infrared metamaterial perfect absorber by combining the dipole, quadrupole, and octopole plasmon resonance modes of four different ring-strip resonators. Opt. Express..

[30] Hu D, Wang H, Tang Z, Zhang X, Zhu Q (2016). Design of four-band terahertz perfect absorber based on a simple #-shaped metamaterial resonator. Appl. Phys. A..

[31] Saadeldin AS, Hameed MFO, Elkaramany EM, Obayya SS (2019). Highly sensitive terahertz metamaterial sensor. IEEE Sens. J..

[32] Shen F, Qin J, Han Z (2019). Planar antenna array as a highly sensitive terahertz sensor. Appl. Opt..

[33] Li Y (2019). Four resonators based high sensitive terahertz metamaterial biosensor used for measuring concentration of protein. J. Phys. D Appl. Phys..

[34] Meng T, Hu D, Zhu Q (2018). Design of a five-band terahertz perfect metamaterial absorber using two resonators. Opt. Commun..

[35] Al-Naib I (2018). Thin-film sensing via fano resonance excitation in symmetric terahertz metamaterials. J. Infrared Millim. Terahertz Waves.

[36] Meng K (2019). Increasing the sensitivity of terahertz split ring resonator metamaterials for dielectric sensing by localized substrate etching. Opt. Express.

[37] Wu X (2021). A four-band and polarization-independent BDS-based tunable absorber with high refractive index sensitivity. Phys. Chem. Chem. Phys..

[38] Zhang R, Zhang R, Wang Z, Li M, Li K (2022). Liquid refractive index sensor based on terahertz metamaterials. Plasmonics.

[39] Wang BX (2022). Miniaturized and actively tunable triple-band terahertz metamaterial absorber using an analogy I-typed resonator. Nanoscale Res. Lett..

[40] Zhan Y, Yin H, Wang J, Yao H, Fan C (2022). Tunable multiple band THz perfect absorber with InSb metamaterial for enhanced sensing application. Results Opt..

[41] Karthikeyan M (2022). Tunable optimal dual band metamaterial absorber for high sensitivity THz refractive index sensing. Nanomaterials.

[42] Islam M, Rao S, Kumar G, Pal BP, Roy Chowdhury D (2017). Role of resonance modes on terahertz metamaterials based thin film sensors. Sci. Rep..

[43] Wang BX, Xu W, Wu Y, Yang Z, Lai S, Lu L (2022). Realization of a multi-band terahertz metamaterial absorber using two identical split rings having opposite opening directions connected by a rectangular patch. Nanoscale Adv..

[44] Zhang Y (2019). A double-band tunable perfect terahertz metamaterial absorber based on Dirac semimetals. Results Phys..

[45] Cheng YZ, Huang ML, Chen HR, Guo ZZ, Mao XS, Gong RZ (2017). Ultrathin six-band polarization-insensitive perfect metamaterial absorber based on a cross-cave patch resonator for terahertz waves. Materials.

[46] Wang BX (2017). Quad-band terahertz metamaterial absorber based on the combining of the dipole and quadrupole resonances of two SRRs. IEEE J. Sel. Top. Quantum Electron..

[47] Wang BX, Xu C, Zhou H, Duan G (2022). Realization of broadband terahertz metamaterial absorber using an anti-symmetric resonator consisting of two mutually perpendicular metallic strips. APL Mater..

[48] Abdulkarim YI (2022). A symmetrical terahertz triple-band metamaterial absorber using a four-capacitance loaded complementary circular split ring resonator and an ultra-thin ZnSe substrate. Symmetry..

[49] Nickpay MR, Danaie M, Shahzadi A (2022). Highly sensitive THz refractive index sensor based on folded split-ring metamaterial graphene resonators. Plasmonics.

[50] Nickpay MR, Danaie M, Shahzadi A (2022). Graphene-based tunable quad-band fan-shaped split-ring metamaterial absorber and refractive index sensor for THz spectrum. Micro Nanostruct..

[51] Anik MHK, Mahmud S, Mahmood KS, Isti MIA, Talukder H, Biswas SK (2022). Numerical investigation of a gear-shaped triple-band perfect terahertz metamaterial absorber as biochemical sensor. IEEE Sens. J..

[52] Li G (2012). A novel plasmonic resonance sensor based on an infrared perfect absorber. J. Phys. D Appl. Phys..

[53] Nickpay MR, Danaie M, Shahzadi A (2023). A triple-band metamaterial graphene-based absorber using rotated split-ring resonators for THz biomedical sensing. Opt. Quant. Electron..

[54] Liu PY (2016). Cell refractive index for cell biology and disease diagnosis: Past, present and future. Lab Chip.

[55] Cong L (2015). Experimental demonstration of ultrasensitive sensing with terahertz metamaterial absorbers: A comparison with the metasurfaces. Appl. Phys. Lett..

[56] Geurts P, Ernst D, Wehenkel L (2006). Extremely randomized trees. Mach. Learn..

[57] Patel SK, Surve J, Katkar V, Parmar J (2022). Machine learning assisted metamaterial-based reconfigurable antenna for low-cost portable electronic devices. Sci. Rep..

[58] Patel SK, Parmar J, Katkar V, Al-Zahrani FA, Ahmed K (2022). Ultra-broadband and polarization-insensitive metasurface absorber with behavior prediction using machine learning. Alex. Eng. J..

